# Early Onset of Heat-Shock Response in Mouse Embryos Revealed by Quantification of Stress-Inducible *hsp70i* RNA

**Published:** 2007-12-06

**Authors:** Cristina Hartshorn, Aleksandra Anshelevich, Yanwei Jia, Lawrence J. Wangh

**Affiliations:** Department of Biology, Brandeis University, Waltham MA 02454-9110, U.S.A

**Keywords:** heat-shock, embryo quality, preimplantation embryo, qRT-PCR, single-tube quantification, stress

## Abstract

Heat shock response is fully established in mouse embryos at the blastocyst stage, but it is unclear when this response first arises during development. To shed light on this question, we used a single-tube method to quantify mRNA levels of the heat shock protein genes *hsp70.1* and *hsp70.3* (*hsp70i*) in individual cleavage-stage embryos that had or had not been heat-shocked. While untreated, healthy embryos contained very low copy numbers of *hsp70i* RNA, heat shock rapidly induced the synthesis of hundreds of *hsp70i* transcripts per blastomere at both the 4-cell and the 8-cell stages. In addition, we performed *hsp70i* measurements in embryos that had not been heat-shocked but had been very slow in developing. Quantification of *hsp70i* RNA and genomic DNA copy numbers in these slow-growing embryos demonstrated the presence of two distinct populations. Some of the embryos contained considerable levels of *hsp70i* RNA, a finding consistent with the hypothesis of endogenous metabolic stress accompanied by cell cycle arrest and delayed development. Other slow-growing embryos contained no *hsp70i* RNA and fewer than expected *hsp70i* gene copies, suggesting the possibility of ongoing apoptosis. In conclusion, this study demonstrates that mouse embryos can activate *hsp70i* expression in response to sub-lethal levels of stress as early as at the 4-cell stage. Our results also indicate that quantification of *hsp70i* DNA and RNA copy numbers may provide a diagnostic tool for embryonic health.

## Introduction

The ability of blastocyst-stage mouse embryos to overcome heat shock (HS) by expression of specific heat shock proteins (HSPs) has been described in numerous studies, particularly focusing on the HSP70 family of stress chaperones (reviewed by Christians et al. 1997a, 2003; De Los Rios et al. 2006). It is now clear that cognate genes belonging to this family, such as *hsc70*, are constitutively expressed during pre-implantation development, while the heat-inducible *hsp70.1* (Hunt and Calderwood, 1990) is spontaneously transcribed only during the very first burst of zygotic genome activation (Christians et al. 1995).

In the past, however, the identification of genes belonging to the *hsp70* family presented some confusion, *hsp70.1* being often designated as *hsp68* (Hahnel et al. 1986; Edwards et al. 1995; Bevilacqua et al. 1995). Increasing knowledge of the mouse genome has recently helped to clarify the precise roles and nomenclature of these genes (reviewed by Christians et al. 2003). In addition, a second heat-inducible gene, *hsp70.3*, has been identified in the mouse genome in close proximity to *hsp70.1* (Huang et al. 2001). The two genes (henceforth called *hsp70i*, Huang et al. 2001; Hartshorn et al. 2005; Singleton and Wischmeyer, 2006) produce proteins that only differ by 22 nucleotides in the regulatory region (Christians et al. 1997a); for these reasons, their individual expression patterns are not known but their roles appear to be similar.

The *hsp70i* proximal 500 bp include a number of regulatory elements essential for the initiation of transcription. Short sequences named heat shock elements are involved in both induced and spontaneous activation of the *hsp70i* promoters (Christians et al. 1997a, 1997b) and bind to the murine heat shock transcription factors mHSF1 and mHSF2 (Mezger et al. 1994; Christians et al. 1997a). Although their functions are not completely known, both of these factors are present in the embryo beginning at the 2-cell stage.

In spite of all this information, whether murine embryos at the cleavage stages can synthesize HSP70 proteins in response to hyperthermia remains a largely unanswered question, although there is growing evidence that this capability is acquired earlier than previously believed. To investigate this possibility, we have measured changes in *hsp70i* RNA levels of control and heat-shocked embryos at different developmental stages employing real-time PCR in combination with the single-tube PurAmp method (Hartshorn et al. 2005). This new technique developed in our laboratory permits quantitative recovery, reverse transcription, and amplification of RNAs down to the level of single cells.

## Materials and Methods

### Embryo culture and HS procedure

Mouse embryos (B6C3F1 females bred with B6D2F1 males) were purchased frozen at the late 2-cell stage or at the compacted 8-cell stage from Embryotech Laboratories, Inc. (Wilmington, MA). Two-cell embryos were used to obtain cleavage stage embryos and were cultured from 1 hour (2-cell stage) to about 20 hours (pre-compaction 8-cell stage) according to a previously described procedure (Hartshorn et al. 2002). Cleavage stage embryos that were delayed several hours in reaching a specific developmental landmark were identified as “slow-growers”. The slow-developing and normally-developing embryos used for this study appeared identical once they had reached either the 4-cell stage or the 8-cell stage, depending on the experiment. Only specimens with even-sized, well-formed blastomeres without fragmentation were analyzed. Slow-growing embryos, however, had reached the desired developmental stage well after the other embryos in the same culture dish. For example, slow-growing 4-cell embryos were identified and harvested from dishes where co-cultured embryos developing at normal rate had already reached the 8-cell or morula stage. Similar criteria were used to identify slow-growing 8-cell embryos. The normal developing rate of the embryos in culture was established based on data collected in this laboratory over five years using the same mouse strain and culture media, and was corroborated by data available in the literature. Blastocyst stage embryos were grown from the embryos purchased at the compacted 8-cell stage and were collected when fully expanded or had began to hatch.

HS was induced by placing the embryos at 43 °C for 30 minutes unless specified otherwise in the text, followed by a recovery period at 37 °C. The duration of the recovery period was variable and is given for each experiment in the Results section. Cell division did not usually occur in these embryos during the recovery time, due to HS-triggered cell cycle arrest, and the number of blastomeres comprising each embryo was therefore unchanged at the time of collection. Control embryos were maintained at 37 °C throughout culture and were harvested without further treatment at stages of development equivalent to those of the heat-shocked embryos (same cell number).

### Sample preparation, reverse transcription (RT) and real-time PCR

In most experiments, embryos collected after HS and recovery as well as control embryos were lysed, reverse transcribed and analyzed by quantitative real-time PCR using the single-tube PurAmp method. A complete description of this protocol and of the composition of the assay for *hsp70i* RNA and DNA measurements is given elsewhere (Hartshorn et al. 2005), but we include here a short summary of the PurAmp strategy. PCR tube lids were pre-loaded with 20-nl droplets of lysing reagents that were allowed to dry. Individual embryos, suspended in a very small volume of buffer, were then delivered directly to the PCR tube lids, where cell lysis occurred very rapidly releasing both RNA and DNA. Samples were quickly dried and could be stored in a freezer until use. Reverse transcription and real-time PCR were then carried out in the same tube by successive dilutions. Initially, a small volume of water was added to the dry samples on the lids. Each tube was closed upside-down and inverted. The content was centrifuged to the bottom and the remaining reagents were added, so that the whole process, from cell lyisis to quantitative PCR, took place in a single vessel.

For the initial experiment carried out with pooled blastocyst-stage embryos (see Results section), nucleic acids were instead extracted and purified in phenol-chloroform using a commercial kit, according to a previously detailed method (Hartshorn et al. 2004, 2005). This procedure will be designated henceforth as the “multistep” template preparation protocol. The purified, pelleted nucleic acids were re-suspended with 6 μl of RT-primer solution, exactly as in the case of the lysed, dried PurAmp samples. From this point onwards every step of the *hsp70i* assay was the same as for the PurAmp-treated embryos.

### *Hsp70i* DNA and RNA copy number quantification

The fluorescent dye SYBR^®^ Green (FMC BioProducts, Rockland, ME) was used to detect the accumulation of amplicons during PCR, as previously described (Hartshorn et al. 2002, 2005). Electrophoresis on 3% (w/v) agarose gel and melting profile analysis (Bernard et al. 1997) confirmed the presence of a single PCR product of the expected size and melting temperature, providing the basis for accurate template number quantification.

Calculation of template copy numbers was based on the “threshold cycle” (C_T_) at which fluorescent signals first arise above background (Walker, 2002). A shift of one cycle between two C_T_ determinations indicates a two-fold difference in the number of amplified templates, with a lower C_T_ value corresponding to an earlier detection of the fluorescent signal and therefore to more templates present at the start of the reaction.

The number of *hsp70i* RNA and genomic DNA copies in each embryo was calculated as detailed elsewhere (Hartshorn et al. 2005). Briefly, samples after RT contained both *hsp70i* cDNA (representative of RNA) and genomic DNA template copies. The cDNA and genomic DNA sequences were amplified simultaneously by a single primer pair and were identical, because the *hsp70i* genes are intronless (Hunt and Calderwood, 1990; Huang et al. 2001). This allowed quantification of cDNA copy numbers by comparison with standard curves obtained with serial dilutions of commercially available genomic DNA (Hartshorn et al. 2002, 2003, 2004, 2005). Levels of *hsp70i* RNA in blastocysts were calculated by measuring genomic DNA-only template numbers in “No RT” samples and subtracting them from the total template numbers of “+RT” samples. In cleavage stage embryos, where cells could be counted, *hsp70i* RNA levels were obtained by subtracting four template copies per cell from total *hsp70i* template numbers, based on the fact that mouse DNA contains four *hsp70i* template copies per genome (one copy of *hsp70.1* and one copy of *hsp70.3* on each chromosome 17).

## Results

**Figure 1:**
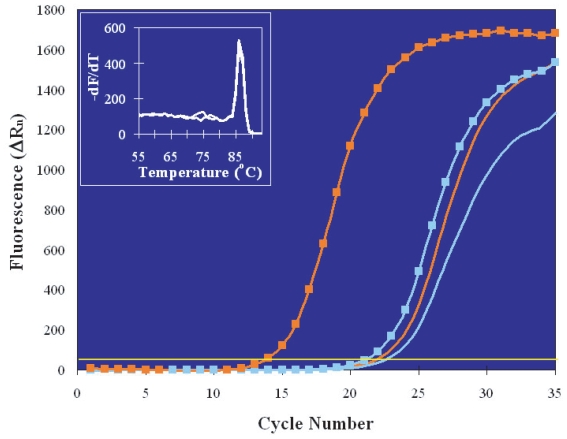
Real-time PCR plots of the amplification of total *hsp70i* templates (cDNA + genomic DNA) in pooled blastocyst stage embryos (five embryos per pool). One embryo pool was not treated and one was heat-shocked for 30 minutes; post-HS recovery also lasted 30 minutes. Each pool was then divided in two aliquots and analyzed with or without RT. From right to left: No HS/No RT, blue line; HS/No RT, orange line; No HS/RT, blue line with squares; HS/RT, orange line with squares. The melting profiles of the heat-shocked embryos PCR products, in the presence or absence of RT, are shown in the inset. They are identical, indicating amplification of the same sequence in cDNA and genomic DNA templates.

Initial experiments aimed at measuring *hsp70i* RNA induction were carried out on pools of embryos at the late blastocyst stage, when HS response is known to be fully established (Christians et al. 1997a). Total nucleic acids were purified using the multistep protocol (see Materials and Methods) and the resulting mixture of RNA and DNA was then divided into two equal aliquots, one of which was subjected to RT prior to PCR amplification. Thus, one aliquot provided a measure of genomic DNA plus cDNA (representative of RNA) *hsp70i* templates, while the other contained only genomic DNA (Hartshorn et al. 2002, 2005). Figure 1 shows the *hsp70i* real-time PCR plots generated by heat-shocked (orange lines) and control (blue lines) embryos in the presence (square markers) or absence (no markers) of RT. Blastocysts maintained at 37 °C contained very little *hsp70i* RNA, since the C_T_ of the reverse transcribed sample was only one and a half thermal cycles earlier than that of the genomic DNA-only sample (compare the blue lines with and without marker), indicating that these embryos had a low level of spontaneous *hsp70i* transcription of the order of 15 copies per genome. (Details on the conversion of C_T_ values into copy numbers and RNA quantification are given in the Materials and Methods section.) The number of genomes measured in the heat-shocked embryo pool (orange line without marker) was about the same as in the control pool. (The slight difference, of about half C_T_, can be explained by the fact that, although the two pools were comprised by equal numbers of embryos, cell number variations are certain to be present among blastocysts and impossible to determine under the microscope because cell boundaries are indistinguishable at this late stage.) In contrast, the number of *hsp70i* RNA copies present after HS increased to 524 per genome, as calculated from the much earlier C_T_ value of the “+RT” signal (orange line with squares). The specificity of PCR amplification was supported by melt profile analysis (inset) that confirmed the presence of a single amplification product with the expected melting temperature in both “+RT” and “No RT” samples.

All the successive experiments were performed with the PurAmp method on single embryos, in order to quantify *hsp70i* expression in preimplantation embryos at different developmental stages and, at the same time, assess the variability of stress response among individual specimens.

We initially monitored the time-course of *hsp70i* RNA accumulation in individual blastocysts placed at 37 °C for a period of time of 30–180 minutes after exposure to 43 °C for 30 minutes. The average number of *hsp70i* RNA copies per embryo increased sharply during the first 60 minutes of post-HS recovery and remained elevated for at least two more hours. However, considerable individual variation was observed among the embryos, including those cultured and heat-shocked together and allowed to recover for at least 60 minutes. Of six blastocysts analyzed under these conditions, three had a much higher *hsp70i* RNA + DNA content (127,293 ± 36,069 copies per embryo, average ± s.d.) than the other three (36,063 ± 3279), suggesting the presence of high and low responders in the population or individual differences in the speed and ability to recover, and thus revert to basal *hsp70i* expression levels.

Additional experiments demonstrated that both the length of the heat treatment and the duration of the recovery are critical for induction of a robust and reproducible response. For instance, one of three blastocysts heated for only 15 minutes and allowed to recover for 30 minutes already displayed elevated *hsp70i* RNA content. On the other hand, only four of six total blastocysts heated for 30 minutes and allowed to recover for 30 minutes in the course of different experiments exhibited an increase in *hsp70i* transcripts above basal levels. In contrast, all blastocysts subjected to a 30-minute HS followed by a recovery period of at least 40 minutes contained elevated levels of *hsp70i* RNA. Based on these results and on parallel analyses on cleavage stage embryos, all subsequent heat treatments were carried out for 30 minutes and were followed by a 2-hour recovery period.

**Figure 2:**
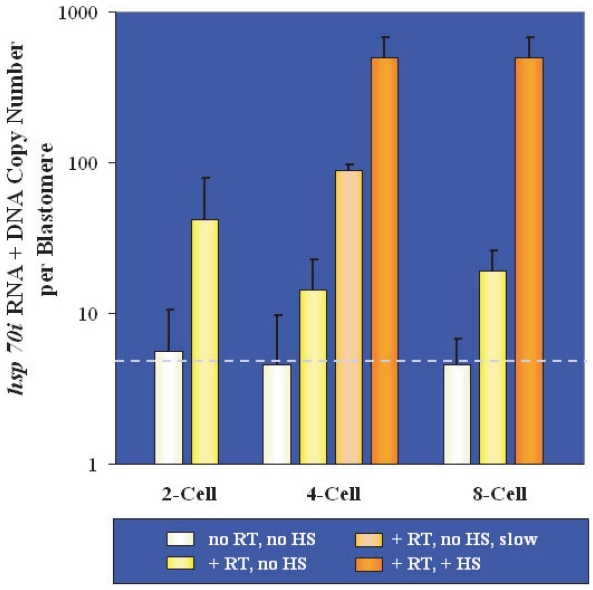
Average numbers of *hsp70i* RNA and DNA molecules measured in cleavage stage embryos. The results of single-embryo determinations were divided by the number of cells comprising the embryo (abscissa) and are presented on a per blastomere basis (black line, s.d.) Normally growing embryos were analyzed after or without HS, in the presence or absence of RT, as shown by the differently colored bars (white, yellow and dark orange; see key in the figure). Slow-developing 4-cell embryos were assayed in the absence of HS but after RT (light orange bar). The average number of *hsp70i* genomic DNA copies per cell is indicated by the dotted horizontal line, which marks 5 copies; *hsp70i* template copies above this line are due to the presence of *hsp70i* RNA.

As a next step we measured *hsp70i* RNA copy numbers in embryos during the cleavage stages of development. Figure 2 summarizes the results of our quantitative analysis of 2-cell, 4-cell and 8-cell stage embryos cultured exclusively at 37 °C or heat-shocked at 43 °C for 30 minutes followed by a 2-hour recovery period at 37 °C. The *hsp70i* RNA + DNA copy numbers in the graph are the averages of several determinations and are plotted on a per blastomere basis, in order to allow comparison between different stages. The numbers of *hsp70i* template copies (RNA + DNA) per cell counted in non-heat-shocked embryos following RT and PCR were: 42 ± 37 (2-cell stage), 14 ± 8 (4-cell stage) and 19 ± 7 (8-cell stage), as shown by the yellow bars. When RT was omitted, the *hsp70i* template copy numbers measured were: 6 ± 5 per cell at the 2-cell stage, 5 ± 5 per cell at the 4-cell stage and 5 ± 2 per cell at the 8-cell stage (white bars), indicating that our assay was quantitatively accurate. Embryonic blastomeres are, in fact, asynchronous and DNA duplication occurs in some cells ahead of others (Graham and Lehtonen, 1979), so that the number of genomes is slightly higher than the number of cells. Based on these considerations, the bar portion above the dotted horizontal line represents *hsp70i* RNA levels, while the bar portion below the line accounts for the presence of an average 5 copies of *hsp70i* genomic DNA per cell. After HS, the average *hsp70i* template numbers per cell (dark orange bars) were 497 ± 177 in 4-cell stage embryos and 499 ± 172 in 8-cell embryos, indicating a considerable increase in *hsp70i* RNA levels compared to non-heat-shocked embryos. A few of the embryos cultured to the 4-cell stage developed slowly and accomplished the second cell division with considerable delay compared to most. These embryos were collected and analyzed separately, in the absence of HS, and turned out to contain an average of 89 ± 9 *hsp70i* RNA + DNA copy numbers (light orange bar), much higher than their healthier counterparts (yellow bar in the same group), potentially indicating cellular stress.

**Figure 3:**
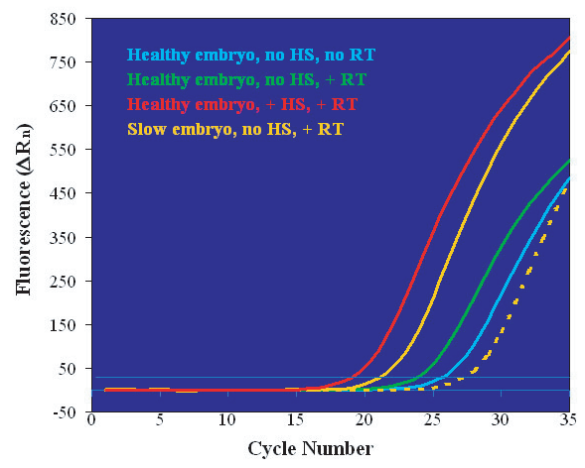
Comparison of *hsp70i* real-time PCR plots obtained from normally-growing (blue, green and red lines) and slow-growing (orange lines) 8-cell embryos. Experimental conditions are given in the figure’s key. Slow-developing embryos fell in two categories, exemplified by the solid line (embryos containing *hsp70i* RNA) and the broken line (embryos undergoing genomic DNA degradation) and detailed in the text.

We further investigated the relationship between embryo quality and *hsp70i* expression levels by monitoring embryos’ growth rate to the 8-cell stage. Five embryos identified as “slow-growing”, although morphologically indistinguishable from the others, were processed in the absence of HS and compared to fifteen normally-developing 8-cell embryos (Fig. 3). For clarity, the figure shows the real-time PCR plots of representative individual embryos. Normally-growing embryos presented a pattern comparable to that described for blastocysts (see Fig. 1), as follows. In the absence of RT only genomic DNA *hsp70i* templates were amplified (blue line); the number of template copies increased slightly after RT due to the presence of low basal levels of *hsp70i* transcripts, as indicated by the earlier C_T_ of the fluorescent signal (green line). HS induced a large accumulation of *hsp70i* RNA molecules corresponding to a bigger shift in C_T_ (red line). The slow-growing embryos fell in two categories, represented by the examples in Figure 3 (solid and broken orange lines). Two of the slow-growing embryos analyzed contained *hsp70i* RNA in levels almost as high as those of the heat-shocked embryos (compare solid orange and red lines). The other three slow-growing embryos had a total *hsp70i* template number lower than the number of genomic *hsp70i* DNA copies (compare broken orange and blue lines), suggesting the possibility that these embryos had initiated apoptosis prior to the appearance of clear morphological changes.

## Discussion

The results of our analyses, carried out on single embryos, are the first direct evidence that cleaving embryos exposed to temperature elevation consistently accumulate thousands of copies of *hsp70i* RNA both at the 4-cell and at the 8-cell stages. In addition, our data show that the number of copies of *hsp70i* RNA per heat-shocked blastomere remains practically unchanged at these two stages, suggesting that embryonic cells resulting from the second and the third division are capable of the same maximal response.

Numerous earlier studies aimed at monitoring the appearance of inducible HSPs in heat-treated preimplantation mouse embryos failed to detect these proteins prior to the blastocyst stage (Morange et al. 1984; Hahnel et al. 1986; Bensaude et al. 1991), possibly due to technical limitations of the methods available at the time. Molecular methods for nucleic acids detection such as the one used for the present investigation are in fact highly sensitive, down to the level of single molecule (Hartshorn et al. 2003, 2005), providing more accurate quantification of gene expression than protein measurements. In addition, a recovery period suitable for gene transcription and protein synthesis in heat-shocked samples was not always included in previously published experiments (reviewed by Christians et al. 1997a). However, synthesis of inducible HSP70 has been observed more recently in 8-cell stage mouse embryos that have undergone compaction (Mezger et al. 1991) and in pre-compaction 8-cell embryos exposed to a low level of hyperthermia (40 ºC) insufficient to trigger HS in blastocysts (Edwards et al. 1995). This second finding parallels the observation that an initial exposure to low levels of heat can induce thermotolerance in 8-cell mouse embryos (Ealy and Hansen, 1994). The fact that such pre-treatment ensures embryos’ survival after severe HS suggests expression of stress chaperone molecules, although it is controversial whether this happens at such an early developmental stage preferentially in embryos developed *in vitro* or *in vivo*. Two studies from the same laboratory generated contrasting results, highlighting the possibility that differences in culture medium composition, HS conditions and mouse strain may play into the experimental outcome, and cautioning against general conclusions (Ealy and Hansen, 1994; Aréchiga and Hansen, 1998). Taken as a whole, the above evidence is consistent with our observation that mouse pre-compaction 8-cell embryos are able to respond to hyperthermia by massively boosting transcription of the heat-inducible genes *hsp70i*. We have further consolidated our data on *hsp70i* HS-response in 8-cell embryos in the course of separate studies carried out at the single blastomere level (Hartshorn et al. 2005).

Embryos at the 4-cell stage present a more complex situation in regard to HSP expression. The prevalent view is that murine embryos at this stage are not able to transcribe HSP genes in response to heat (Mezger et al. 1994; Bevilacqua et al. 1995), although embryos of other species, such as bovine, can do so as early as at the 2-cell stage (Edwards et al. 1997). Christians et al. (1995) have also shown, using both RT-PCR and a transgenic line of *HSP70.1Luc* (*hsp70.1* promoter coupled to the firefly luciferase reporter) mice, that *hsp70.1* is spontaneously expressed very early in development but is then rapidly silenced. This very first burst of zygotic transcription peaks in the 2-cell stage before completion of the second round of DNA replication and is almost completely downregulated by the 4-cell stage.

The reasons for this reported lack of HSP70s synthesis, either spontaneous or induced, at the 4-cell stage are not clear and have been the focus of intensive investigation. Some evidence suggests that the *hsp70i* transcription machinery can fully function at this stage and there is no shortage or sequestration of available transcription factors, in spite of a likely decline in maternally transcribed molecules. In fact, microinjection of *HSP70.1Luc* plasmids in 2-cell stage embryos induced transient expression at the 4-cell stage (Christians et al. 1995). Similarly, when a construct containing the *hsp70*.1 promoter and the *lacZ* reporter gene was injected into 4-cell embryos as an episome, transcription was activated, although not in a HS-dependent manner (Bevilacqua et al. 1995). In contrast, when either the *HSP70.1Luc* or the *HSP70.1lacZ* construct were integrated into the genome the promoter activity declined at the time of the second cleavage. Based on this observation the authors concluded that intragenic or transacting repressors must normally silence the *hsp70i* genes at this particular time in development. Transient changes in chromatin structure have also been considered as possible reasons for a temporary inactivation of the endogenous *hsp70.1* promoter.

More recent analyses by Christians et al. (1997a, 1997b), however, clearly demonstrate that the *HSP70.1Luc* promoter is activated by HS at all stages of preimplantation development. This careful study compares activity levels from the 2-cell stage to the blastocyst stage by accounting for differences in the number of cells comprising the embryos, and also investigates the time-course of transcription activation. Christians’ results show that the speed of response to HS changes dramatically between the morula and the blastocyst stage, being much faster in blastocyst than in cleavage stage embryos, but that maximal activity is very high at all developmental stages. Although the significance of the slower HS-dependent transcription activation at early stages is still unclear and leaves many questions open (Christians et al. 1997a), these findings are generally in agreement with our measurements of *hsp70i* RNA accumulation in 4-cell-to-blastocyst stage embryos and with our observation that, while most blastocysts accumulate *hsp70i* transcripts within 30 minutes from the end of HS, this recovery time is insufficient for expression in 8-cell embryos and 32-cell morulas (not shown). The results of the present study thus provide strong evidence for the view that stress-induced *hsp70i* expression in mouse embryos begins at the 4-to-8 cell stage (Luft and Dix, 1999), even though it is possible that the transcription rate of this response increases in blastocysts.

Our quantification of spontaneous *hsp70i* expression in 2-cell embryos shows the presence of a number of RNA molecules about four times higher than in embryos of later stages. While this ratio appears lower than reported by others (Christians et al. 1995), it is actually comparable when considering that we used embryos at the late 2-cell stage while non-inducible *hsp70i* expression peaks very early, before DNA replication in 2-cell blastomeres, and that culturing conditions greatly affect the extent of this expression. Embryos grown under the culture conditions described in this paper develop at a rate similar to embryos developed *in vivo* (Wang et al. 2004) and are therefore likely to have low levels of spontaneous *hsp70i* expression at the 2-cell stage (Christians et al. 1995). In addition, the extent of zygotic *hsp70i* expression has been shown to be strain-specific and very variable (Chastant et al. 1996). As a final consideration, we used embryos that had been frozen at the 2-cell stage, a procedure that may lead to loss of some of the RNA initially present. All these factors led us to conclude that our measurements were accurate at all stages of development, in the presence or absence of HS.

The fundamental role of HSPs in allowing embryos to survive harmful environmental conditions has been widely investigated (reviewed by Luft and Dix, 1999; Christians et al. 2003). Based on this knowledge, it was not surprising that cleaving embryos growing sub-optimally contained relatively high levels of *hsp70i* RNA. It could be speculated that those slow-developing specimens that did not contain *hsp70i* RNA had initiated apoptosis. The HSP70i are in fact known anti-apoptotic factors necessary for protection against oxidative stress (Luft and Dix, 1999; Huang et al. 2001; Christians et al. 2003), although their function at very early stages of development is not precisely understood, as shown by the fact that heat-shocked 2-cell embryos are irreversibly blocked and do not progress through the cell cycle in spite of active spontaneous *hsp70i* transcription. Our data, however, suggest that finely tuned quantitative analyses of *hsp70i* expression levels could potentially be indicative of embryonic health at all stages and, if performed in single blastomeres, could have diagnostic applications in human and animal *in vitro* fertilization settings.
